# Evaluation of Taste Acceptance of Three Different Fluoride Varnishes in Children with Autistic Spectrum Disorder: A Randomized Clinical Trial

**DOI:** 10.3390/jcm14061948

**Published:** 2025-03-13

**Authors:** Rohini Mohan, Guna Shekhar Madiraju, Chiew Ying Chieng, Yousef Majed Almugla, Faris Yahya I. Asiri

**Affiliations:** 1 Pediatric Dentistry, Community Dental Service, Swansea Bay University Health Board, Port Talbot Resource Centre, Port Talbot SA12 7BJ, UK; 2 Department of Preventive Dental Sciences, College of Dentistry, King Faisal University, AlAhsa 31982, Saudi Arabia; yalmugla@kfu.edu.sa (Y.M.A.); fasiri@kfu.edu.sa (F.Y.I.A.); 3 Queens Hospital, Rom Valley Way, Romford RM7 0AG, UK

**Keywords:** child, autism spectrum disorder, fluoride varnish, taste, prevention, behavior

## Abstract

**Background/Objective**: The taste perception of clinical materials used in dental treatment procedures can influence the compliance of autistic children during dental visits due to their heightened anxiety and sensory processing difficulties. This study aimed to evaluate the taste acceptance of different fluoride varnish preparations among children with autism spectrum disorder (ASD) in a clinical setting. **Methods**: This parallel-arm single-blinded randomized clinical study included autistic children aged 6–14 years, referred to a community dental clinic unit for preventive dental care. Non-verbal behavior, as a measure of taste acceptance, was assessed using the Frankl behavior rating scale. Additionally, subjective taste responses were recorded using a 3-point facial hedonic scale. Data were analyzed using descriptive statistics and the chi-square test. **Results**: There was no statistically significant difference in non-verbal behavior between the three fluoride varnish groups before application (*p* = 0.094) or immediately after application (*p* = 0.718). However, when comparing pre- and post-application responses within each group, Duraphat^®^ showed a significant improvement in non-verbal behavior (*p* = 0.020), while no significant changes were observed for Profluorid^®^ (*p* = 0.196) or MI Varnish^®^ (*p* = 0.704). Subjective taste acceptance, as measured by the 3-point facial hedonic scale, showed no significant differences among the varnish groups (*p* = 0.406). **Conclusions**: Flavored fluoride varnishes may improve the compliance of autistic children with preventive oral care procedures. Although no significant differences in taste acceptance were observed among the three varnishes, Duraphat^®^ was associated with a significant improvement in non-verbal positive behavior, suggesting a more favorable response in autistic children.

## 1. Introduction

Autism spectrum disorder (ASD) is a neurological development disorder where affected individuals display a broad range of characteristics that can affect their ability to communicate, learn, and behave. They find it harder to process new sensations due to their sensory processing differences and are more likely to be hyper- or hyposensitive [1]. Autistic patients can be extremely sensitive to certain external stimuli and taste, such as mouth rinse or the feel of dental instruments in oral cavity. They tend to get distressed when dental clinicians get close to them during the course of appointments [1,2]. In addition, children affected with ASD generally have poor tongue coordination and therefore prefer a sweetened, soft diet, which is held in their mouths for prolonged duration instead of swallowing, therefore resulting in increased caries risk [3]. This combined with tooth brushing difficulties, lack of coordination, and sensitivity to taste and smells might more likely make autistic children seek dental treatment for decay [3,4]. Hence, preventive measures in these children has been advocated to reduce the likelihood of the development of dental caries [2]. Fluoride varnishes aid in preventing caries through high fluoride concentration and a prolonged contact time with tooth surfaces. The effectiveness of fluoride varnishes for controlling caries has been clearly demonstrated in the literature [5,6]. While fluoride varnish provides an effective option for caries prevention in patients with special needs, various formulations of fluoride varnishes, available in different concentrations, have been introduced and reported to demonstrate clinical efficacy in preventing caries [7].

Alterations in taste sensitivity have been reported in children with ASD [8]. Despite having normal detection thresholds, they often exhibit diminished ability to identify and differentiate between tastes [8,9]. The taste perception of dental materials might be an important factor to determine the compliance of autistic children to dental clinical procedures. To date, little is known about the effect of autistic traits on behavior related to sensory information processing, including smell and taste perception. While previous studies have mostly investigated the efficacy of fluoride varnishes on caries prevention in children, no studies are available on the taste acceptance of different fluoride preparations in children with ASD. Accordingly, the present study aimed to evaluate the taste acceptance of three different fluoride varnish preparations among children with autism spectrum disorder in a clinical setting. The research hypothesis posited that there would be a statistically significant difference in taste acceptance among the three different FV preparations.

## 2. Materials and Methods

This study was designed as a single-center, single-blinded, randomized clinical trial (RCT) with parallel-group design and was conducted in accordance with the Consolidated Standards of Reporting Trials (CONSORT) guidelines [10]. This was a single-blinded study, where the principal researcher applying fluoride varnish was aware of the intervention, but the participants, outcome assessors or examiners, and data analysts were not aware of the varnish used and study outcomes in order to minimize ascertainment bias.

### 2.1. Sample Size

A sample size of 20 individuals per FV group was calculated to be sufficient based on crude chi-square tests with dichotomous outcomes based on an expected 40% difference (effect size) between the three FV preparations. The final required sample size was 60 participants for 5% alpha error and 80% power with 95% level of confidence [11]. As no similar study with relevant effects was found, this was considered a clinically relevant effect. The calculations did not take into account the use of the ordinal Likert scale in the analyses or the fact that no patients received the three products in a parallel design.

### 2.2. Ethical Aspects

The study was approved by the Review and Ethical committee, Swansea Bay University Health Board, UK (Ref: 19/WA/0033), and was carried out in compliance with the Helsinki Declaration. Written informed consents were obtained from the children’ parent/legal guardian in both English and Welsh language, using an interpreter where needed.

### 2.3. Study Setting

Sixty-five autistic children aged 6 to 14 years, who were referred for dental care at the community dental clinics of Port Talbot Resource Centre, Port Talbot, Wales, United Kingdom, were recruited and invited to participate in the study, during the period from January to November 2023. A trained dental nurse was in charge of enrolling patients who met eligibility criteria, and the project leader (RM) carefully assessed and selected the final 60 eligible patients to participate in the study. The inclusion criteria were children diagnosed with ASDs aged between 6–14 years at the time of oral examination, children with previous or current caries experience who were indicated for FV application based on caries risk assessment (CAMBRA), and written informed consent signed by parent/guardian. The exclusion criteria were coexistence of diseases that may affect taste assessment (type 1 diabetes, infectious diseases requiring antibiotic therapy, neoplastic diseases, etc.), oral thrush and injuries of the oral mucosa, ulcerative gingival stomatitis, lack of informed consent, voluntary withdrawal of the parent or legal guardian at any stage of the procedure, previous hospital admission for uncontrolled asthma, history of dental care provided in sedation or general anesthesia, and known sensitivity to colophony [12] (Duraphat^®^, Profluorid^®^) and milk protein or hydroxybenzoates allergy (MI Varnish^®^) [13].

### 2.4. Study Design

The taste acceptance of the fluoride varnish compounds Duraphat^®^ (Colgate, New York, NY, USA), Profluorid^®^ (VOCO GmbH, Cuxhaven, Germany), and MI Varnish^®^ (GC Europe N.V., Leuven, Belgium) were investigated. Duraphat varnish is a yellowish suspension and contains 5% sodium fluoride (NaF) by weight, and the main ingredients are natural resins such as colophonium, mastix, and shellac in an alcoholic suspension (pH 7; banana flavor). MI Varnish is a bioavailable varnish preparation that contains 5% NaF and 2% Recaldent™ (GC America Inc., Alsip, IL, USA) (Casein Phosphopeptide-Amorphous Calcium Phosphate, CPP-ACP) dispersed in a rosin and ethanol solution (pH 6.6; strawberry flavor). Profluorid varnish is an ethanolic suspension of colophony with artificial flavors and sweetened with xylitol (pH 6.6; cherry flavor). The three FV preparations were presented without labels because of their similar application methods. Frankl behavior rating scale was used to measure the subjects’ non-verbal behavior (objective data) as a measure of taste acceptance of three different fluoride varnishes, and a 3-point facial hedonic scale [14] (subjective data) was used to assess a subject’s acceptance of the preparation. The primary outcome of interest was the negative or positive non-verbal behavior measured using Frankl behavior rating scale.

### 2.5. Clinical Procedure

**Figure 1:**
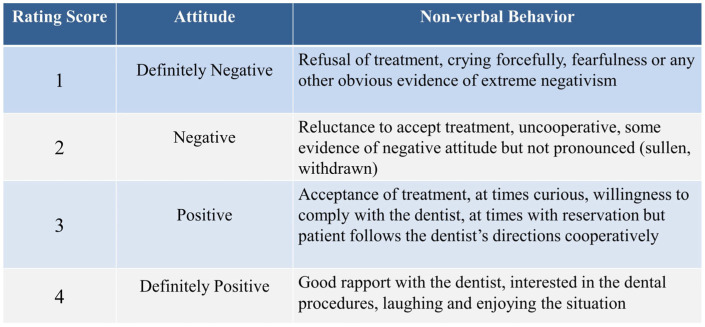
Frankl behavior rating scale used to collect objective feedback.

The intervention consisted of three phases preceded by an initial phase where the procedure was explained and demonstrated to children in a standard dialogue by the project leader (RM) using the tell-show-do technique for behavior management. Later, they were instructed and guided on how to indicate the taste of the FV preparation on the three-point facial hedonic scale, which included faces with a laughing, crying, or neutral facial expression [14]. The time period from a child’s entry into the operating room up to the FV application, i.e., before application, included phase I, while phase II consisted of the time period immediately after FV application. The non-verbal behavior of each of the subjects was assessed before (phase I) and immediately after the FV application (phase II) by using the Frankl behavior rating scale [15], ranging from “definitely negative” to “definitely positive” (Figure 1). The nonverbal behavior of each subject before fluoride varnish application was evaluated by the project leader (RM) and was blinded to the allocation of participants. The dental nurse assigned the participants to the three FV interventions randomly with an allocation ratio of 1:1:1. A simple random allocation method was used where each participant was randomly allocated to one of the three groups as they arrived. This method ensured that each participant had an equal chance of being assigned to any of the groups and was concealed until the exact moment of assignment. Twenty participants were included in each of the three groups to allow for comparison of the three flavored fluoride varnishes. The first group received the banana-flavored Duraphat varnish, the second group received strawberry-flavored MI Varnish, and the third group was applied with cherry-flavored Profluorid varnish. The tell-show-do behavior management technique was used for all the participants [16]. FV applications were performed by the project leader, who is a trained pediatric dental specialist (RM), with microbrushes (Hager & Werken GmbH & Co., Ltd., Duisburg, Germany) in age-appropriate dosages, following the manufacturer’s instructions.

**Figure 2:**
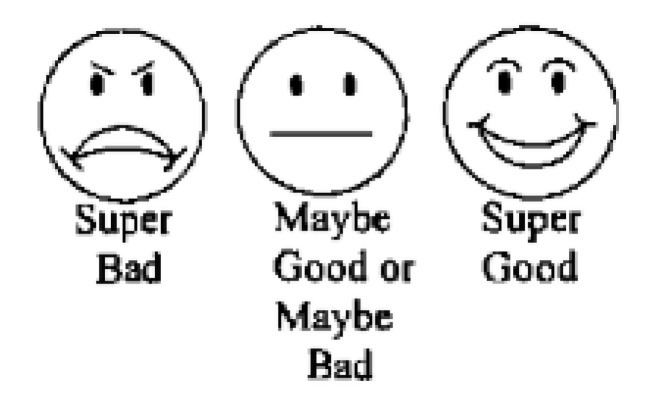
Subjective feedback from children collected using 3-point facial hedonic scale.

Two trained calibrated dentists who were blinded to the type of FV preparation and outcomes of the study examined and evaluated the nonverbal behavior of each subject immediately after application using the Frankl behavior rating scale. The mean score of the two examiners who rated the behavior was recorded for each child, and the intraclass correlation coefficient (ICC) used to measure interrater reliability was found to be 0.88. After FV application, each child was additionally asked to choose the appropriate smiley face from the 3-point hedonic scale (phase III) (Figure 2) that closely represented their evaluation of the taste of FV preparation they received, while another interviewer (CC), who was blinded to the type of varnish used and study outcomes, recorded the feedback from subjects.

### 2.6. Statistical Analysis

Data were entered in Microsoft Excel and analyzed using the Statistical Package for the Social Sciences (SPSS) (version 20.0, SPSS Inc., Chicago, IL, USA). Descriptive statistics and frequencies were ascertained. Categorical variables for the two groups were com-pared using Pearson’s chi-square test. Nonverbal behavior immediately after fluoride varnish application was recorded as a measure of taste acceptance of the three fluoride varnishes. Additionally, the acceptance of FV preparation by the children was subjectively assessed using a 3-point facial scale. The chi-square test was used to investigate the effect of three different fluoride varnish preparations on the taste acceptance of children with autistic traits. The normality tests (Kolmogorov–Smirnov and Shapiro–Wilk) were used to assess whether the data followed a normal distribution. The relationship between the variables, age, and behavior response outcomes was assessed using correlation analysis. Data were analyzed at a 95% confident interval with significance level set at 0.05.

## 3. Results

**Figure 3:**
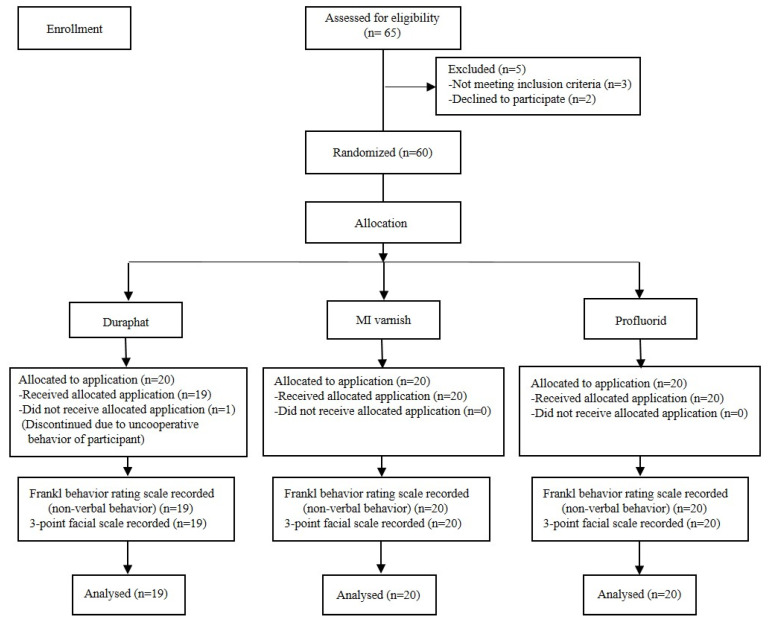
Flow diagram of participants and different phases of the study according to CONSORT 2010 guidelines.

A total of 59 out of 60 children underwent FV application; one child was excluded, as he refused the application despite initial behavior management and abstained from the procedure (Figure 3). So, the final sample for analysis consisted of 59 children (53 boys and 6 girls) aged 6–14 years, while the mean age of the study sample was 9.64 ± 3.25 (mean ± SD). Regarding the assessment of non-verbal behavior of participants by the Frankl behavior rating scale, the values of the rating scale were dichotomized with score values 1 and 2 as “negative” and values 3 and 4 as “positive” for data analysis purposes.

There were no significant differences in the non-verbal behavior of subjects allocated to the three varnish groups before the FV application (χ^2^ = 4.70; *p* = 0.094). To evaluate the taste acceptance immediately after application, the non-verbal behavior of the subjects analyzed using chi-square test for all the three FV groups demonstrated that the effect of FV taste on children’s nonverbal behavior was not significant (χ^2^ = 0.663; *p* = 0.718; n = 59). When the nonverbal behavior was compared within each of the varnish groups before and after the application, Duraphat showed a significant effect on the change in behavior, with more children responding positively or accepting the taste of Duraphat (χ^2^ = 5.397; *p* = 0.020). However, no significant changes in non-verbal behavior were noted within the Profluorid (χ^2^ = 1.666; *p* = 0.196) and MI Varnish application groups (χ^2^ = 0.143; *p* = 0.704), even though there were some noticeable improvement in subjects with positive behavior responses (Table 1).

The non-verbal behavior of children scored based on Frankl behavior rating scale showed no significant differences between the three FV groups, with respect to being either positive or negative. Among 26 children who showed non-verbal negative behavior before varnish application, 12 of them presented positive response after the application. The remaining 14 children who had displayed negative behavior in phase I refused fluoride varnish application, thereby repeating the negative behavior, which was closely reflected in similar responses from the smiley ratings (super bad, n = 13). Children who presented prior positive behavior (phase I) displayed significantly more positive responses after the varnish application (phase II). There was no significant effect of the varnish preparations on the taste acceptance of children based on their feedback from three-point smiley ratings (χ^2^ = 3.998; df = 4; n = 59; *p* = 0.406) (Table 2). Due to non-normal distribution, Spearman’s correlation was performed, which showed no significant relationship between the participants age and either pre-intervention (r = −0.033; *p* = 0.803) or post-intervention behavior responses (r = −0.190; *p* = 0.148).

## 4. Discussion

Children’s behavioral responses during dental procedures needs consideration, as anxiety and fear are prevalent and can profoundly influence their overall experience and level of cooperation [17]. Since preventive care is more frequently delivered to children, the understanding of their behavior during such sessions is important. Children with ASD are usually more anxious in the dental office, and the type of response or behavior elicited by them is unknown or inadequate [18]. Moreover, these children tend to communicate in a nonverbal manner while receiving preventive care such as FV application in clinical settings [19]. Studies in the literature have revealed the relationship between autistic traits and taste preferences or perceptions [20]. However, there are no reports about the taste acceptance of different flavored fluoride varnishes in children with ASD. Only one study conducted by Kolb et al. [21] examined the taste acceptance of highly concentrated fluoride preparations in a kindergarten-based preventive program and concluded that the use of flavors in fluoride preparations results in higher acceptance among preschool children. The present study is the first to investigate the taste acceptance of different FV preparations in children with autistic traits within a clinical setting. The nonverbal behavior immediately after application was rated as an objective measure of taste acceptance, while the 3-point facial hedonic scale was used as the subjective measure. The analysis revealed no significant differences in taste acceptance outcomes between the three FV preparations, nor was there a significant association between taste acceptance and autistic status within the varnish groups. So, in this regard, the research hypothesis of the current study was partially rejected.

Previous studies have recommended the use of flavored compounds to enhance the acceptance of preventive care procedures [21]. All the three fluoride varnishes used in the present study had fruity flavors and neutral pH ranging from 6.6 (MI Varnish^®^, Voco Profluorid^®^) to 7.0 (Duraphat^®^). In general, children who present positive cooperative behavior will usually accept the FV application compared to those who display negative behavior. In this study, other than the subjects with positive behavior, 12 subjects (20.4%) with negative behavior before the FV application had later displayed positive behavior after the application. This may be due to the pleasant and flavored taste of the FV applied to them. Only 14 subjects (23.7%) exhibited negative behavior and refused varnish application even with use of the tell-show-do behavior management technique. The possible role of successful behavior management using the tell-show-do technique should not be overlooked, as this could contribute to the increase in positive behavior in children during and after the FV application.

Children over the age of six years may find it easier to associate their taste perceptions with symbolic facial expressions. Therefore, this study focused on children aged 6–14 years and employed a 3-point facial hedonic scale to assess taste acceptance of FV in children with autism. Facial scales use illustrations of various facial expressions to represent different sensory experiences, eliminating the need for the child to quantify their experiences numerically [22]. These scales are particularly useful in cognitively impaired children, as they require less cognitive effort to express preferences for foods [13]. However, a limitation of facial hedonic scales involving smiley faces is their inability to detect subtle differences in the symbolic expression of taste perception, which is particularly important when working with children with ASD. For objective data, the Frankl behavior rating scale is considered more reliable and is frequently used in both clinical dentistry and research. It has been shown to provide more accurate assessments than facial smiley scales or visual analog scales [23]. The Frankl scale aids in measuring nonverbal behaviors, which are often the primary response to gustatory stimuli in children [21]. Fluoride varnish application has been recommended based on risk assessment, which includes factors such as previous or current caries experience. In the present study, all the recruited children were classified as being at moderate to high caries risk. Balian et al. [6] recommended early dental examinations for children with ASD and suggested routine application of fluoride varnishes and dental sealants, regardless of caries risk, age, or level of cooperation, as a more effective strategy for reducing caries risk.

To the best of our knowledge, this study is the first to assess the behavioral responses of children with ASD undergoing preventive dental interventions in a clinical setting. In this observational study, non-verbal behavior was used as an indicator of taste acceptance for three different fluoride varnishes. The findings revealed no significant differences in the non-verbal behavior of autistic children following the application of the three fluoride varnishes, which implied that the children accepted all three varnishes similarly. While each varnish resulted in improvement in positive behavior responses, Duraphat was the only varnish to demonstrate a statistically significant change. In contrast, Profluorid and MI Varnish showed improvements but were not statistically significant, which implied that their effect might not be as strong as Duraphat in this context. However, the potential influence of the flavor of the fluoride varnish preparation on taste perception should also be considered. Moreover, the inclusion of flavoring agents in the FV preparations may affect the rankings, as these agents can mask the original taste of the active ingredient. Therefore, individual taste preferences should be considered when interpreting the findings.

The significance of this study lies in its novelty, as there are no prior studies comparing different types of flavored fluoride varnishes in terms of taste acceptance (sensory) while also examining their impact on the non-verbal behavior of children with ASD. Duraphat varnish is the most widely used FV preparation in the United Kingdom and is the only product licensed as a prescription-only medicine specifically for caries prevention. The greater efficacy of Duraphat in the present study could possibly be attributed to its unique formulation, which appears to be more effective in managing behavioral responses in this population. This study demonstrated that the MI and Profluorid varnish applications were equally accepted, although the results were statistically non-significant compared to Duraphat with regard to taste acceptance in our study population. Since this study showed a comparable taste acceptance for all the three varnishes measured at the objective (non-verbal behavior) and subjective (3-point hedonic scale) levels, the possibility of including varnishes other than Duraphat should be considered for application in children, taking into account the additional benefits of CCP-ACP in MI Varnish or the cost effectiveness of other varnishes. This is especially true of NHS practices that do not have a choice in fee setting. However, limitations of each of the FV studied, such as colophony allergy [12] related to Duraphat and Profluorid and milk protein allergy related to MI Varnish [13], should be taken into consideration before selection of FV for application in autistic children. Marinho et al. [24] suggested that prescription of fluoride varnishes other than Duraphat that are off-label and not licensed for caries control or prevention needs proper consideration in the best interests of the patient, although they may contain similar formulations.

### 4.1. Limitations

The findings drawn from this study should be interpreted in the light of a few limitations. As previously mentioned, the limitation in sample size may restrict the generalizability of these findings to the broader population of autistic children. Additionally, the inability to stratify results by age due to this limited sample size prevented the assessment of age-related differences and may have introduced age as a confounding factor. However, the results of this trial may be applicable to children within similar groups and settings. Additionally, this study predominantly included male participants, and due to limited research on this topic, direct comparisons with other studies may be challenging. The DSM-5 (Diagnostic and Statistical Manual of Mental Disorders, Fifth Edition) has combined all autism diagnoses into a single diagnosis of ASD but with three different levels for support needs. The three ASD levels of severity include level 1 (“requiring support”), level 2 (“requiring substantial support”), and level 3 (“requiring very substantial support”) [25]. In the present study, all participants with autism were identified as having either level 1 or level 2 autism spectrum disorder, according to the DSM-5 criteria. Furthermore, the specific type of autism spectrum disorder was not considered and hence not included as a variable in this study. This limitation should be acknowledged when interpreting the study’s findings.

### 4.2. Future Research Directions

Future studies should aim to include larger, more diverse samples to enable meaningful stratification by age, allowing for a more comprehensive understanding and improving the generalizability of the findings. Inclusion of both male and female participants will help identify potential gender-based differences in taste acceptance and behavioral responses. Additionally, categorizing participants based on autism spectrum severity levels may offer valuable insights into variations in acceptance and behavior. Furthermore, future research should also explore the role of familiarization protocols (e.g., pre-exposure to varnish taste) in enhancing compliance and acceptance among children with autism.

## 5. Conclusions

This study provides valuable insights into the taste acceptance of different flavored fluoride varnishes among children with autism spectrum disorder (ASD) and their impact on non-verbal behavior during preventive dental care. The findings indicate that all three fluoride varnishes, Duraphat^®^, Profluorid^®^, and MI Varnish^®^, were well accepted, with no statistically significant differences in taste acceptance among them. While Duraphat fluoride varnish demonstrated the most significant improvements in positive behaviors, both Profluorid and MI Varnish also showed improvements, albeit not statistically significant. Given the comparable outcomes observed across all three varnishes, Profluorid and MI Varnish may also be considered viable alternatives for preventive dental care in children with autism. This is particularly pertinent considering the added benefit of CCP-ACP in MI Varnish and the cost effectiveness of Profluorid and MI Varnish relative to Duraphat, especially in the context of NHS dental services. Although there is no definitive evidence to suggest that one varnish is superior to the others, the findings suggest that flavored fluoride varnishes in general may enhance compliance with preventive dental care, particularly for children with sensory sensitivities. Further clinical investigation is needed to confirm these findings and guide future recommendations.

## Figures and Tables

**Table 1:** Non-verbal behavior of subjects based on Frankl behavior rating scale.

Varnish Used	Frankl Behavior Rating Scale				*p*-Value ^††^
	Before Application		After Application		
	Positive *n* (%)	Negative *n* (%)	Positive *n* (%)	Negative *n* (%)	
Duraphat^®^	08 (42.1%)	11 (57.9%)	15 (78.9%)	04 (21.1%)	0.02 (χ^2^ = 5.397)
MI Varnish^®^	15 (75%)	05 (25%)	16 (80%)	04 (20%)	0.704 (χ^2^ = 0.143)
Profluorid^®^	10 (50%)	10 (50%)	14 (70%)	06 (30%)	0.196 (χ^2^ = 1.666)
*p*-value ^†^	*p* = 0.094 (χ^2^ = 4.7)		*p* = 0.717 (χ^2^ = 0.663)		

Chi-square (χ^2^) test; *p*-value ^†^ is between groups; *p*-value ^††^ is within groups.

**Table 2:** Non-verbal behavior (taste acceptance) of subjects based on smiley ratings.

Varnish Used	3-Point Facial Hedonic Scale n (%)			Chi-Square (χ^2^) Test *p*-Value
	Super Good	Maybe Good or Maybe Bad	Super Bad	
Duraphat^®^	10 (52.6%)	6 (31.6%)	3 (15.8%)	0.406 (χ^2^ = 3.998)
MI Varnish^®^	10 (50.0%)	4 (20.0%)	6 (30.0%)	
Profluorid^®^	14 (70.0%)	2 (10.0%)	4 (20.0%)	
Total	34	12	13	59

## Data Availability

The data presented in this study are available on reasonable request from the corresponding author.
